# Analysis of the legislative process related to the implementation of graphic health warning labels on tobacco products in South Korea

**DOI:** 10.18332/tid/115035

**Published:** 2020-01-16

**Authors:** Ji-eun Hwang, Sung-il Cho, Sun-goo Lee

**Affiliations:** 1 Institute of Health and Environment, Seoul National University, Seoul, Republic of Korea; 2 Department of Public Health Sciences, Graduate School of Public Health, Seoul National University, Seoul, Republic of Korea; 3 Underwood International College, Yonsei University, Seoul, Republic of Korea

**Keywords:** tobacco control, graphic health warning labels, legislative process, tobacco company, Framework Convention on Tobacco Control (FCTC)

## Abstract

**INTRODUCTION:**

Well-designed health warnings on tobacco packaging enhance cost-effectively public awareness of the risks of using tobacco products. However, many countries have experienced difficulties in implementing pictorial warnings. The purpose of this study is to present the topics that arose during the legislative process that preceded implementation of graphic health warning labels (GHWLs) on tobacco products in South Korea, and discuss the outcomes.

**METHODS:**

We used qualitative content analysis to analyze lawmakers’ statements, and those of committee members in meetings that preceded the drafting of the legislative document pertaining to GHWLs in South Korea.

**RESULTS:**

In discussions surrounding the adoption of the GHWLs, the main point of contention was the level of disgust induced by pictorial warnings. When discussing how warnings should be inscribed on packaging after adoption of GHWLs, lawmakers disagreed regarding the physical position of the warnings. Because of continuous objections raised by some lawmakers, implementation of GHWLs was delayed, and, when actually introduced, the warnings were toned down. Some lawmakers communicated with tobacco companies; thus the companies participated in the legislative process in South Korea.

**CONCLUSIONS:**

To prevent tobacco companies from negatively influencing tobacco control efforts, it is essential that all communications with such companies be publicly disclosed and that the tobacco industry be prohibited from contacting lawmakers involved in the legislative process of tobacco control.

## INTRODUCTION

Article 11 of the Framework Convention on Tobacco Control (FCTC), and the guidelines thereof, emphasize that well-designed health warnings on tobacco packaging enhance cost-effectively public awareness of the risks of using tobacco products^1,2^. In particular, Article 11 states that tobacco packaging should include warning text and pictures or pictograms that cover 50% or more of the principal display area, and that relevant parties are obliged to comply within 3 years of the convention’s ratification^1^. To date, however, only 64% of the relevant parties have adopted pictorial warnings^3^.

In South Korea, graphic health warning labels (GHWLs) were implemented 23 December 2016^4^, although South Korea ratified the FCTC in 2005^5^. Since 2002, bills proposing revisions to the National Health Promotion Act (NHPA) (thus requiring the implementation of GHWLs) were routinely abandoned without any deliberation in the National Assembly of Korea (‘the Assembly’)^5^; for a period exceeding 10 years, the bills died in committees without ever being debated.

It is important to analyze the legislative process when seeking to implement policies; and it is necessary to anticipate and deal with barriers and challenges^6^. Worldwide, the methods by which the tobacco industry opposes tobacco control are similar; an analysis thereof will facilitate the establishment of tobacco control policies^7^.

Here, we describe the topics raised in the legislative debates that preceded the implementation of GHWLs in South Korea, and their outcomes. We explore whether the tobacco industry had interfered in the legislative process, and suggest strategies that could lower barriers to the strengthening of tobacco control policies in South Korea and elsewhere.

Current South Korean laws concerning tobacco control include the Tobacco Business Act and the NHPA^8^. The NHPA is concerned with the details of tobacco control, including health warnings on tobacco products^8^; the Tobacco Business Act was designed to improve the contributions of the tobacco industry to the national economy by formalizing tobacco production and distribution^9^. We explored only the process of NHPA revision, as achieved via the Enforcement Decree of the National Health Promotion Act (EDNHPA), which contains the technical details of GHWL enforcement by the NHPA^10^.

The National Assembly Act and Regulations on Management of Legislative Affairs^11,12^ describe how the legislative process in South Korea involves multiple steps, including the drafting of a bill, consulting with relevant organizations, pre-publication of the bill, examination of the bill by the Legislation and Judiciary Committee (LJC) (an Assembly body that reviews the legal wording of all bills)^11^, deliberation and resolution of the bill by the National Assembly, and, finally, promulgation of the bill (https://elaw.klri.re.kr/eng_service/step.do)^13^. On the other hand, an Enforcement Decree does not require deliberation by the Assembly. Instead, the regulations are examined by the Regulatory Reform Committee (RRC), which is composed of both members of the government and civilians^13^. We focused on the revision of two laws concerning GHWLs: the NHPA and ENDHPA. We qualitatively analyzed the content of legislative documents produced by the lawmakers.

## METHODS

The study is divided into two parts, by data source (Table 1). When analyzing the NHPA, we focused on Assembly minutes that recorded the proceedings of plenary sessions or committees. We obtained the minutes of the 15th (1995–2000) to 19th (2012–2016) Assemblies, thus over the period during which the Assembly deliberated adoption of the GHWLs (the NHPA was enacted in 1995 and GHWLs were implemented in 2016). We searched the Bill Information System (http://likms.assembly.go.kr/bill/main.do) using the keywords ‘National Health Promotion Act’^14^. For each bill, the system includes the reports and minutes of every committee and plenary meeting. We identified 192 amendment bills and selected 13 related to GHWLs, excluding those increasing tax on tobacco products and expanding smoke-free areas, among others. We then collected 15 meeting minutes related to the selected bills: 10 from the Health and Welfare Committee, 3 from the LJC, and 2 from the Assembly plenary session.

**Table 1:** Data sources and analytical methods used in this study

*Study part*	*Purpose*	*Data source*	*Time coverage*	*Data*				*Analysis*
				*Type*	*Contents*	*Collected*	*Selected*	
1	To analyze the legislative process related to the implementation of GHWLs	Obtained from the database of the Bill Information System	From 30 May 2000 to 29 May 2016	Amendment bills	DateName of proposersPurpose and content of amendment bill	192	13	Organize the content of the draft bills temporally
				Minutes	Recorded all proceedings of the plenary session or committee meetings	15	3	Categorization of sentences by themes
2	To analyze the legislative process that determined the technical details of GHWLs	Obtained from the Ministry of Health and Welfare and the Korea Health Promotion Institute	From 22 April to 13 May 2016	Meeting documents	Recorded the agenda for discussion	2	2	Summarize and interpret the decisions resulting from the meetings
				Meeting minutes	Recorded the major discussion points and decision results	2	2	

* GHWLs: graphic health warning labels.

Text related to GHWLs was extracted from the minutes and classified as either negative or positive in terms of GHWL implementation. Positive text included arguments in favor of GHWL implementation, such as support for the original plan of the Ministry of Health and Welfare (MOHW). Text opposing tobacco control or requesting weakening of the original draft was considered negative. Using an inductive approach, the first author selected relevant sentences, grouped them, and developed preliminary themes. After repeating this process, all authors discussed and agreed on the final overall themes and translated relevant text from Korean to English.

In terms of the EDNHPA, we analyzed documents associated with the RRC, including meeting agendas and minutes; these recorded the major discussion points and final committee decisions. During the legislative process dealing with the technical details of GHWLs, the RRC twice (on 22 April and 13 May 2016) examined revised EDNHPA bills prepared by the MOHW. The minutes of both meetings were obtained from the MOHW and the Korea Health Promotion Institute. The first author identified key issues, and all authors reviewed and discussed the texts.

## RESULTS

### History of the legislative process related to implementation of GHWLs in South Korea

**Figure 1:**
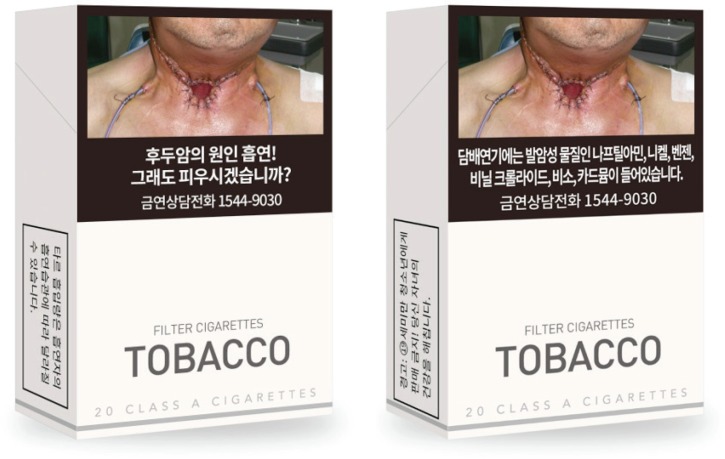
The graphic health warning label placed on the front (left) and back (right) of cigarette packs in South Korea. Front: ‘Smoking causes laryngeal cancer! Still want to smoke? Quit Line 1544-9030’; Left side: ‘The intake of tar varies depending upon a smoker’s smoking habits’; Back: ‘Cigarette smoke contains carcinogenic substances, including naphthylamine, nickel, benzene, vinyl chloride, arsenic, and cadmium’. Quit Line 1544-9030’; Right side: ‘Warning: It is illegal to sell cigarettes to people under 19! It is harmful to your children’s health’

A bill requesting legal incorporation of GHWLs was submitted 12 times (10 times by Assembly members, once by the MOHW, and once by the Health and Welfare Committee of the Assembly) during the period between the 16th Assembly (from 30 May 2000 to 29 May 2004) and the 19th Assembly (from 30 May 2012 to 29 May 2016) (Supplementary file). After three rounds of examination by a committee of the LJC, the final bill was adopted at an Assembly Plenary session on 29 May 2015 and the law came into effect on 23 December 2016 (Figure 1).

### Level of disgust induced by pictorial warnings

LJC member comments opposing implementation of GHWLs were coded into four themes: 1) Insult to smokers and violation of smokers’ rights to pursue happiness; 2) Violation of the rights of the tobacco industry; 3) Protection of tobacco farmers; and 4) Strengthening of mass media campaigns rather than use of GHWLs (Table 2). One LJC member requested that the pictures not be overly graphic, or in photographic form, and that the size of the image be reduced from 30% to 20% of the principal packaging display area. Next, the allegations of infringement on smokers’ rights and protection of their right to pursue happiness were the main topics for debate. Several members who attended the LJC meeting alleged that the pictorial warnings could infringe on fundamental rights, such as those of smokers, if the graphic nature of the images was too disgusting. Indeed, some members insisted that the graphic nature of the pictorial warnings must be limited by a proviso, a condition or limitation included in the legislative documents.

**Table 2:** Quotations from, and themes discussed by, lawmakers obtained from National Assembly records regarding the legislative process for implementing graphic health warning labels in South Korea

*Theme*	*Arguments of the lawmakers during the legislative process in South Korea*
Insult to smokers and violation of smokers’ right to pursue happiness by GHWLs	1st meeting of the 332nd session of the Legislation and Judiciary Committee (1 May 2015) Lee A: ‘Most pictorial warnings show diseased or swollen lung cancers or laryngeal cancerous tumors. Smokers will continue to look at these pictures when smoking, which is believed to be insulting to the smoker. (So, this debate happened this morning.) When smokers look at horrible photos of diseased lungs, it infringes on their rights. The Ministry of Health and Welfare may think this is a strange position, but it is almost intimidating; it intimidates smokers. So, I think that this is absolutely necessary to consider, at least to the extent that we do not infringe the rights of smokers... The disgusting warnings violate smokers’ rights as indicated in the Constitution…’. Kim: ‘The sale of cigarettes is permitted by law. Since smokers are also citizens, shouldn’t we acknowledge smokers’ right to pursue happiness, in line with the constitution?’. 3rd meeting of the 332nd session of the Legislation and Judiciary Committee (6 May 2015) Kim: ‘I would like to say once again that I have made this suggestion in view of the constitution because disgusting images, which are really hateful, may violate the personal rights of smokers’. Lim: ‘Considering smokers, it is possible to limit their rights for health purposes, but it is a constitutional problem in that it violates their essential rights’. Lee B: ‘A health warning can convey that smoking is bad for you. If smoking is bad and the risk to society is serious, would it not be better to stop selling cigarettes?’.
Violation of rights of the tobacco industry by GHWLs	1st meeting of the 332nd session of the Legislation and Judiciary Committee (1 May 2015) Lim: ‘Considering KT&G and the tobacco farmers’ perspective, I think that the graphic warning labels violate the freedom of expression of the tobacco company’.
Protection of tobacco farmers	1st meeting of the 332nd session of the Legislation and Judiciary Committee (1 May 2015) Lim: ‘I received data from KT&G today. After reviewing it, I thought that, if the bill passed, the circumstances for tobacco growers would become more difficult’. Seo: ‘I realized that KT&G, the cigarette distributors, and the tobacco growers are all interrelated’.
Strengthen mass media campaigns instead of GHWLs	1st meeting of the 332nd session of the Legislation and Judiciary Committee (1 May 2015) Seo: ‘To promote healthy practices, various campaigns should be strengthened by the Health and Welfare Committee. I think that a good campaign alone would be enough, without the need for warnings’.

* GHWLs: graphic health warning labels

Some lawmakers argued that tobacco products are legally manufactured and sold under the Tobacco Business Act, so the rights of businesses and tobacco farmers should also be protected. There was also an opinion that tobacco consumption should be reduced in a healthy manner, such as through mass media campaigns and education.

Others claimed that this proviso clause, i.e. that the warning picture should be based on facts without being overly obnoxious, should not be included in the law because it is against the purpose of the Act. Moreover, as it is difficult to measure feelings objectively, it was asserted that ‘overly obnoxious’ is not an appropriate legal term.

Although the warning size reduction was not accepted, the final version of the NHPA included the proviso clause limiting the expression of pictorial warnings to reflect the proposal of the LJC members.

### Interactions between tobacco companies and lawmakers

During the LJC meeting of 1 May 2015, a member of the LJC (Lawmaker Lim) stated: ‘Today, I received data from KT&G. After I read it, I thought that, if the bill passed, the circumstances for tobacco growers would become more difficult’. As interactions between lawmakers and tobacco companies (including KT&G, South Korea’s largest tobacco company) lack transparency, it is difficult to determine how the tobacco company contacted the lawmaker or what information was conveyed. However, it is possible that the company contacted a lawmaker to present the company view, or that of tobacco farmers. The member in question continued to consistently oppose implementation of GHWLs.

### The positioning of pictorial warnings at the top of packaging

The original plan by the MOHW was to locate the pictorial warnings at the top of cigarette packs (Table 3). However, to block this plan, groups supporting the tobacco industry, including the Korea Tobacco Association, the Korea Tobacco Sales Association, the Korea Convenience Store Industry Association, and a smokers group called ‘I Love Smoking’, organized a strong objection by submitting their opinions to the MOHW after the advance publication of the draft of the EDNHPA. Their arguments in the first meeting document were as follows: *‘Because GHWLs limit the design, which is governed by the authority of tobacco manufacturers, the top positioning of pictorial warnings on tobacco products violates the principle of proportionality. And consumers may therefore find it difficult to select the tobacco products’.* At the first RRC meeting, some members also recommended that the requirement of positioning pictures at the top of packaging should be removed and the GHWLs be positioned at the bottom of cigarette packs.

**Table 3:** Comparison of measures actually implemented and the original plan of the Enforcement Decree of the National Health Promotion Act in terms of graphic health warning labels

*Category*	*Original plan*	*Implementation*
**Layout**	Display pictorial warning in a line of rectangles	-
**Placement**	Top	Top
**Font**	Gothic type	Gothic type
**Font color**	Complementary color	Complementary color
**Border**	Bland and 2 mm	Bland and 2 mm
**Rotation**	18 months	24 months
**Prohibition**	Hide the pictorial warning	-
**Number of pictures**	Fewer than 10	Fewer than 10
**Announcement**	Six months before enforcement	Six months before enforcement
**Other**	Each pictorial warning to have the same dimensions	Each pictorial warning to have the same dimensions

However, the MOHW requested a re-examination of this issue and submitted additional documents in support of positioning the GHWLs at the top. As part of this re-examination, the Korea Health Promotion Institute, on behalf of the MOHW, conducted an experimental study to evaluate differences in eye fixation according to the position of the warning images using an eye-tracker device^15^. The results showed that participants focused more on the upper than the lower position^15^. Finally, the RRC accepted that the GHWLs would be positioned at the top.

The RRC proposed two additional recommendations. First, the time until changes had to be made to the GHWLs was extended from 18 to 24 months. Second, the RRC recommended that the paragraph prohibiting the retailer from covering the pictures at the point-of-sale be removed. These were eventually reflected in the final EDNHPA, while the plan to position at the top remained unchanged (Table 3).

### Qualifications of lawmakers participating in the legislative process

The minutes of the first RRC meeting at which the EDNHPA was discussed show that the committee included two members, Suh and Son, representing the tobacco industry: one had served as a non-executive director of KT&G and the other had worked as an advisor to the law office of Philip Morris, which is currently in litigation with the National Health Insurance Corporation. These members stated that the scientific evidence supporting the effectiveness of positioning a pictorial warning at the top was insufficient. Additionally, they argued that placing a pictorial warning at the top of cigarette packets would force store owners to re-install shelves that hid the pictures, a process associated with replacement costs. The RRC thus recommended that the GHWLs be positioned at the bottom of the cigarette packs. However, these two members did not attend the RRC re-examination.

## DISCUSSION

Use of GHWLs represents a scientifically proven, cost-effective tobacco control policy that does not involve pricing; GHWLs are a means of direct regulation of the tobacco industry itself rather than smokers, and can increase the efficacy of anti-smoking policies without the need for public sector spending^16^. Despite their proven efficacy, and the efforts of the MOHW and some members of the Assembly to implement GHWLs, it took 14 years before they were finally implemented after first being proposed at the National Assembly in South Korea. Because of continued objections by certain lawmakers, the policy was delayed and subsequently weakened.

Although rare, connections between the tobacco industry and certain lawmakers were identified in South Korea. In particular, persons directly or indirectly involved with tobacco companies served as ‘civilians’ examining the law concerning GHWLs. Furthermore, some lawmakers’ statements were almost identical to the arguments that the tobacco industry had previously used in its attempts to block the implementation of GHWLs^17^: *Graphic warnings ‘demonize’ smokers; Large graphic health warnings violate tobacco manufacturers’ rights to property, including trade mark protection; If the government wants to disseminate health-warning messages, it should use billboards or TV commercials.* These situations may indicate that tobacco companies had made direct or indirect contact with some lawmakers, where such contact may have influenced lawmakers’ decisions in the legislative process.

Given these opinions, the original plan was modified to weaken the effect of GHWLs during the legislative process. These outcomes also aligned with the tobacco industry’s strategy to obstruct the legislation related to tobacco control, by substituting the industry’s proposal in place of the original plan^7^. This may indicate that the tobacco industry’s influence on the legislative process reduced the efficacy of the GHWLs in South Korea.

However, there is insufficient evidence regarding the means by which the tobacco industry exerted an inappropriate influence on lawmakers. Although the tobacco industry undertakes lobbying to increase its profits by influencing the legislative process^18^, it is difficult to identify tactics that clearly attest to the inappropriate influence that it is believed to exert on lawmakers^19^. According to Article 5.3 of the FCTC, tobacco industry workers and representatives should not be involved in any organization or committee involved in policy formulation and the implementation process, in the interest of protecting tobacco control policies from commercial and other vested interests in the tobacco industry^1^. However, not only supporting groups, which include a tobacco growers’ association, a smokers’ rights group, and tobacco retailing, advertising, law firms^20^ and some compromised lawmakers, were able to participate in the legislative process in South Korea, despite the fact that they had strong interests in the tobacco industry.

Other countries have experienced delays and difficulties in GHWL implementation, caused principally by tobacco industry interference (litigation, lobbying for support from lawmakers and governments, and the use of front groups)^20^. The international tobacco industry has sought to reduce the size of warning labels^21^, to limit the number of colors used^22^, and to render the pictorial warnings ineffective^23^. The industry has also asserted that the images violate freedom of expression, trademark property rights^24^ and even freedom of religion^23^. All of these tactics have delayed implementation of tobacco control policies and undermined their effectiveness^25,26^. In India, GHWL implementation was delayed by both international and domestic tobacco companies^27^; GHWLs have yet to be implemented in the United States because of lawsuits filed by the tobacco industry^28^.

In South Korea, the industry has sought to weaken GHWLs by limiting the images used and reducing their size; the suggestion is that the images and warnings violate smokers’ fundamental rights. As the right to smoke is a limitable constitutional right^29^, any tobacco control regulations may be nationally implemented under the FCTC, as well as by state and local governments, which have implemented such regulations to improve the health of their citizens. In the 2003 Hun-Ma457 case^30^, the Constitutional Court of Korea acknowledged that both the right to smoke cigarettes freely and the right to avoid smoking are constitutional rights. The right to smoke freely is recognized on the basis of human dignity, the right to pursue happiness under Article 10 of the Constitution, and the right to privacy under Article 17 of the Constitution^31^. The Court stressed that the right to avoid cigarette smoking, which is the right of the non-smokers not to smoke and to be free from cigarette smoking, is also based upon Articles 10 and 17 of the Constitution, and the constitutionally guaranteed right to health and right to life, in the sense that the health and life of non-smokers who are exposed to passive cigarette smoking are endangered.

Because both rights are constitutional rights, when they are in conflict—when a policy promotes one right while restricting the other—the Constitutional Court should decide whether such a restriction is constitutional. The Court noted that the right to avoid cigarette smoking is based not only upon the right to privacy but also upon the right to life, which is the premise of all basic rights and occupies the highest position. Therefore, the Court stated, the right to avoid cigarette smoking is a basic right that is prioritized over the right to smoke cigarettes.

To combat this, a system by which the media and community can monitor all lawmakers and members of committees and organizations involved in the legislative process of tobacco control should be established and made accessible by the public^32^. Furthermore, any information provided by the tobacco industry, and any interactions between lawmakers/committee members and the industry, should be disclosed^33^. Individual lawmakers should be obligated to disclose any relationship with a tobacco company^34^.

We found that timely research on implementation of tobacco control policies was useful. As indicated by the data showing the effectiveness of positioning health warnings on the upper part of the cigarette pack, scientific investigation plays a key role in overturning unsubstantiated opinions^24,35^. When plain packaging was implemented in Australia, the tobacco industry argued that this would lead to longer transaction times and customer frustration^36^. However, no increases in pack transaction times were observed (Carter et al.^37^, Bayly et al.^36^, and Wakefield et al.^38^). Additionally, tobacco companies argued that cigarette advertising and display at the point-of-sale target only adult smokers^39^. In fact, frequent exposure to tobacco advertising is associated with higher levels of cigarette brand awareness, and increased rates of smoking initiation^40,41^ and susceptibility among youth^42,43^. Therefore, public health advocates should consistently provide scientific evidence that may be brought to bear upon the regulatory process, and should pressure policymakers to swiftly adopt regulations^44,45^.

### Limitations

This study has several limitations. First, insufficient evidence of a direct connection between the tobacco industry and lawmakers was uncovered. Numerous previous studies have proven the tobacco industry’s use of tactics aimed at delaying and weakening tobacco control policies, by analyzing documents originating from the tobacco industry^27,46,47^. In this study, however, only the minutes and meeting documents were analyzed. During the qualitative content analysis of the minutes, we found no evidence of monetary donations, or donations of any other type, to lawmakers from the tobacco industry. To supplement the study’s main findings, we recommend further analysis of tobacco industry documents in a follow-up investigation. Second, the materials subjected to analysis were not sufficient. To overcome the limitations of such data, several studies have analyzed the contents of media reports^48,49^, since data not considered previously can be extracted from media reports. Third, this study dealt only with the implementation of GHWLs. If future studies were performed examining other tobacco control issues, including tobacco taxation and the designation of non-smoking areas, more of the tobacco companies’ activities, and the political impact thereof, could be identified. Consequently, an expansion in the scope of research in this area is required.

## CONCLUSIONS

The implementation of GHWLs was delayed and weakened due to continued objections by certain lawmakers during the legislative process. These lawmakers had direct or indirect relationships with tobacco companies but nonetheless participated in the GHWL legislative process. However, the commitment of the government (e.g. the MOHW) and a range of research studies, played an important role in refuting oppositional arguments. To promote policies aimed at strengthening tobacco control regulation in the future, it will be necessary not only to prevent any direct or indirect participation on the part of tobacco companies in the legislative process but also to provide a method of monitoring these activities.
