# Supplementary material for: Analysis of the legislative process related to the implementation of graphic health warning labels on tobacco products in South Korea
Source: Tob Induc Dis. 2020 Jan 16;18:03. doi: 10.18332/tid/115035 (PMC6970237; doi:10.18332/tid/115035)
Supplement: Supplementary file 1 [file TID-18-03-s1.pdf]

Appendix A. The history of the legislative progress related to the implementation of graphic health warning labels in South Korea

National Assembly Session	Date	Legislative process and its results
16th	November 18, 2002	A draft of the National Health Promotion Act requiring graphic health warning labels was submitted by 52 Assembly members including Geun-jin Lee, but was invalidated.
	September 25, 2003	A draft of the Act was submitted by 100 Assembly members including Geun-jin Lee, but was invalidated.
17th	February 2, 2007	A draft of the Act was submitted by 16 Assembly members including Jae-cheon Choi, but was invalidated.
	October 2, 2007	A draft of the Act was submitted by the Government of South Korea, but was invalidated.
18th	July 28, 2008	A draft of the Act was submitted by 10 Assembly members including Myung-soo Lee, but was invalidated.
	July 30, 2008	A draft of the Act was submitted by 10 Assembly members including Hong-jun Ahn, but was invalidated.
	November 10, 2008	A draft of the Act was submitted by 10 Assembly members including Hyun-hee Jeon, but was invalidated.
	September 14, 2009	A draft of the Act was submitted by 11 Assembly members including Hye-sook Jeon, but was invalidated.
19th	September 17, 2012	A draft of the Act was submitted by 11 Assembly members including Dae-sung Moon, but was invalidated.
	January 10, 2013	A draft of the Act was submitted by 10 Assembly members including Hong-jun Ahn, but was invalidated.
	March 18, 2013	A draft of the Act was submitted by 10 Assembly members including Jae-won Kim, but was invalidated.
	September 22, 2014	A draft of the Act was submitted by the Government of South Korea.
	February 26, 2015	A draft of the Act was examined by the Health and Welfare Committee of the Assembly, and passed.
	March 3, 2015	The agenda related to the proposed Act requiring graphic health warning labels was submitted to the Legislation and Judiciary Committee.
	May 1, 2015	The agenda related to the proposed Act was submitted to the Legislation and Judiciary Sub-Committee and examined.
	May 6, 2015	The agenda related to the proposed Act was examined by the Legislation and Judiciary Committee and passed.
	May 6, 2015	A draft of the Act was submitted by the Health and Welfare chairperson and passed.
	May 29, 2015	The agenda related to the proposed Act was deliberated and resolved by the Assembly Plenary session.
	June 22, 2015	The final amended Act was promulgated by publication by the Ministry of Health and Welfare.
	October 14, 2015 to November 23, 2015	The proposed Enforcement Decree of the National Health Promotion Act was published by the Ministry of Health and Welfare.
20th	April 22, 2016	The proposed Decree was examined by the Regulatory Reform Commission.
	May 13, 2016	The proposed Decree was re-examined by the Regulatory Reform Commission.
	June 21, 2016	The final amended Decree was promulgated by publication by the Ministry of Health and Welfare.
	June 22, 2016	The public was notified of the final graphic health warning labels (pictures and phase) by the Ministry of Health and Welfare.
	December 23, 2016	The graphic health warning labels were implemented.

© 2020 Hwang J. et al.
